# Gastrointestinal Symptoms Impact Psychosocial Function and Quality of Life in Patients with Rheumatoid Arthritis and Spondyloarthritis: A Cross-Sectional Study

**DOI:** 10.3390/jcm12093248

**Published:** 2023-05-01

**Authors:** Francesco Salvatore Iaquinta, Daniele Mauro, Ilenia Pantano, Saverio Naty, Daniela Iacono, Emanuela Gaggiano, Luca Riccio, Francesco Ciccia, Rosa Daniela Grembiale, Rocco Spagnuolo

**Affiliations:** 1 Department of Health Sciences, “Magna Græcia” University of Catanzaro, 88100 Catanzaro, Italy; saverio_naty@yahoo.it (S.N.); rdgrembiale@unicz.it (R.D.G.); 2 Department of Precision Medicine, University of Campania “L. Vanvitelli”, 80138 Naples, Italy; dranielmar@gmail.com (D.M.); ilenia.pantano@libero.it (I.P.); daniela.iacono@unicampania.it (D.I.); emanuela-gaggiano@libero.it (E.G.); lucariccio1991@gmail.com (L.R.); francesco.ciccia@unicampania.it (F.C.)

**Keywords:** mood disorders, inflammatory arthritis, gastrointestinal symptoms, PROMIS^®^, rheumatoid arthritis, spondyloarthritis

## Abstract

Patients with chronic Inflammatory Arthritis (IA), such as Rheumatoid Arthritis (RA) and Spondyloarthritis (SpA) are more likely to experience psychosocial impairment. Gastrointestinal (GI) symptoms are also present, especially in Spondyloarthritis. No data are available on the relationship between gut and brain manifestations and their impact on daily activities in this setting; thus, this study aimed to assess these symptoms in an IA population and identify potential associations. IA patients and a control group were enrolled. The Patient-Reported Outcome Measurement Instrument System (PROMIS^®^) questionnaire was used to evaluate GI and psychosocial domains. The study included 389 subjects (238 controls and 151 with IA); demographic and clinical data were collected for each participant. IA patients reported both higher psychosocial and GI impairment compared with controls. The logistic regression model revealed a strong association between depression and belly pain (*p* = 0.035), diarrhea (*p* = 0.017), bloating (*p* = 0.018), and reflux (*p* = 0.01); anxiety was associated with belly pain (*p* = 0.004), diarrhea (*p* = 0.019), swallowing alterations (*p* = 0.004), flatulence (*p* < 0.001) and reflux (*p* = 0.008). Moreover, fatigue, sleep disorders, and pain interference were associated with almost all GI symptoms, whereas high physical function scores and satisfaction in social roles decreased the odds of most GI symptoms. IA patients had more significant impairment in both dimensions compared with controls. To address reported symptoms and improve the overall quality of life in rheumatologic patients, a new holistic approach is required.

## 1. Introduction

The umbrella term “chronic inflammatory arthritis (IA)” refers to a heterogeneous group of rheumatic diseases, including Rheumatoid Arthritis (RA), and peripheral and axial Spondyloarthritis (SpA), all characterized mainly by joint pain and inflammation. A dysregulated immune system is thought to be the key feature of the disease in genetically predisposed individuals [1,2]; however, etiopathogenesis has not been entirely elucidated. The contribution of intestinal dysbiosis and subclinical gut inflammation is emerging in both RA and SpA [3,4].

In this regard, it is broadly accepted that patients with RA and SpA experience more frequently mood disorders, mainly anxiety and depressive symptoms, leading to impairment of daily activities [5]. Under the current definition of health by the World Health Organization (WHO) as a state of complete physical, mental, and social well-being, mood disorders may significantly impact the health status of IA patients [6,7,8,9]. This is also confirmed by the worse patient global assessment score observed in patients with RA and concomitant gastrointestinal (GI) symptoms (64%) [10]. The high frequency of GI symptoms could have a biological basis, including the side effect of treatment and the involvement of the GI system in the pathogenesis of IA.

Intestinal dysbiosis and subclinical gut inflammation have been demonstrated in both RA and SpA and it may contribute to the pathogenesis of these diseases [2,3,4,11,12,13,14,15,16,17]. More recently, it has been hypothesized that intestinal dysbiosis and possibly aberrant gut permeability may be involved in the development of mood disorders suggesting the existence of a gut–brain axis [18,19,20,21].

However, in this setting, no clinical research has yet investigated the potential relationship between gut inflammation or microbiome alteration and mood disorders and the impact on quality of life.

Quality of life is commonly assessed through patient-reported outcomes measures (PROMs) that consist of a set of predetermined validated questions measuring self-reported outcomes, such as disease perception and health-related quality of life [22]. For this purpose, the Patient-Reported Outcomes Measurement Information System (PROMIS^®^), a set of extensively validated and standardized item banks, represents good support for the assessment of both psychosocial (anxiety and depression, sleep disturbances, pain, social life satisfaction) [23,24], and GI symptoms [25].

So far, little evidence is available on the correlation between mood disorders and gastrointestinal manifestations and how these affect the quality of life in IA patients. Hence, in this study, we aimed to define the prevalence of both psychosocial and gastrointestinal symptoms, assessed by PROMIS^®^, in a cohort of patients with the most common chronic IA (RA and SpA) compared with a control population, trying to identify potential relationships between these symptoms.

## 2. Materials and Methods

### 2.1. Study Participants

Between March 2021 and August 2021, consecutive outpatients attending the Rheumatology Units of the “Magna Græcia” University of Catanzaro and “Luigi Vanvitelli”, the University of Naples, and a control group, that is volunteers recruited outside of the clinic, were enrolled in the study. Controls have been selected from the general population and the same geographical area of the patients, matched by age. Participants aged ≥18 years and able to give informed consent were enrolled. The following have been considered as exclusion criteria for both patients and controls: (a) non-Italian speaking subjects; (b) people unable to read or understand the survey or with cognitive impairment; (c) patients previously diagnosed with mood disorders, gastrointestinal immune-mediated inflammatory diseases such as inflammatory bowel diseases (IBD) and celiac disease; histologically proved gastritis, colitis or esophagitis, radiologic and/or endoscopic GERD (d) controls with autoimmune or autoinflammatory diseases.

All patients had a confirmed diagnosis of RA, Psoriatic Arthritis (PsA), and SpA, according to EULAR/ACR [26], CASPAR [27], and ASAS [28] classification criteria, respectively. Demographic (age, sex, smoking habit, employment status, educational level, marital status) and anthropometric (Body Mass Index (BMI)) details were obtained for each subject. Moreover, participants were asked if they did any physical activity and the question has been scored positive if a threshold of a minimum of 30 min at least twice a week was met. Information on treatment, i.e., biologic agents, steroids and disease-modifying anti-rheumatic drugs (DMARDs), disease duration, C-reactive protein (CRP), and erythrocyte sedimentation rate (ESR) levels, were also collected. Patients underwent a thorough evaluation of disease characteristics, such as disease activity assessed with DAS28 (CRP) for RA (with active disease defined by scores > 2.6) [29]; DAPSA28 (CRP) used for PsA (values > 4 identified the disease as active) [30]; and ASDAS for axial SpA (with scores >1.3 defining active disease) [31] (Supplementary Table S1).

### 2.2. Ethics Committee Approval

This study was approved by the ethics committee of “Magna Græcia” University (protocol 69/2018). All the procedures were conducted in accordance with the Declaration of Helsinki principles and its amendments. Each participant provided written informed consent.

### 2.3. Questionnaires

Participants were invited to complete a survey on psychosocial dimensions and gastrointestinal symptoms through PROMIS^®^ items (v1.0) (https://www.promishealth.org/ (accessed on 1 October 2018) while waiting for the scheduled visit. A trained physician instructed subjects to fill in the form without any influence from healthcare professionals.

Psychological and social functioning included the assessment of anxiety (Short Form 8a), depression (Short Form 8a), sleep disturbances (Short Form 8a), pain interference (Short Form 8a), fatigue (Short Form 8a), physical function (Short Form 8b) and satisfaction with participation in social roles (Short Form 8a) through the Italian versions available. To note, the PROMIS^®^ Physical Function item investigates the self-reported capability of physical activities (including walking/mobility and dexterity) and instrumental activities of daily living, such as running errands or carrying groceries.

Since no current translations of PROMIS^®^-GI are available, the English versions were used. To avoid bias in understanding, for participants not able to understand the exact meaning of the questions, these were translated by the same authors (F.S.I. in Catanzaro and D.M. in Naples). PROMIS^®^-GI scales assessed seven domains: gastroesophageal reflux (13a), constipation (9a), disrupted swallowing (7a), diarrhea (6a), nausea and vomiting (4a), belly pain (5a), and gas/bloating (13a). Regarding gastroesophageal reflux, the scale assesses symptoms associated with stomach contents leaking backwards into the esophagus and the frequency of regurgitation, the experience of burning or a lump in the throat, burping, hiccupping, and excessive saliva production. However, the measure is not disease-specific, therefore, is not referred exclusively to gastroesophageal reflux disease (GERD).

The PROMIS^®^ item bank employs a five-point Likert scale that ranges from 1 (Never/Not at all) to 5 (Very much/Always). The survey investigated symptoms and their interference with daily activity in the past seven days. PROMIS^®^ scores are normalized on a t-score metric based on the general U.S. population, with a mean of 50 and a 10-point standard deviation. For positive-worded items (Physical function and satisfaction with participation in social roles), a higher t-score indicates a better condition. Conversely, a higher t-score indicates a worse condition for the rest of the negative-worded items. A t-score cut-off of 50 was used to dichotomize the total scores for each domain. Specifically, t-scores of 50 or higher suggest the presence of a clinically significant condition, and t-scores below 50 indicate its absence.

### 2.4. Data Analysis

A Kolmogorov–Smirnov test was carried out to determine the distribution of variables. Data are shown as median with interquartile range (IQR) for continuous variables. Categorical variables are presented as frequencies and percentages. Statistical analysis was conducted to identify differences among groups in the demographic, anthropometric characteristics and domains studied, using a non-parametric approach. The Mann–Whitney U and Chi-squared tests were performed as appropriate. Moreover, a binary logistic regression model, adjusted for age, sex, BMI, smoking habit, non-steroidal anti-inflammatory drugs (NSAID) (yes/no), steroids (yes/no), and type of IA was conducted to determine the psychosocial variables associated with GI symptoms in the IA group. Logistic regression results are expressed as odds ratio (OR) with a 95% confidence interval (95% CI). IBM SPSS^®^ Statistics (IBM, Armonk, NY, USA), Version 26 was used to conduct the analysis. A *p*-value ≤ 0.05 was considered to be statistically significant.

## 3. Results

### 3.1. Characteristics of the Study Cohort

Table 1 shows the enrolled subjects’ demographic, anthropometric, and clinical characteristics. In detail, 389 individuals were involved in the study: 238 (61.2%) controls and 151 (38.8%) with IA (*n* = 47, 31.1% RA and *n* = 104, 68.9% SpA). A significant sex difference (*p* = 4 × 10^−6^) and BMI (*p* = 1.18 × 10^−4^); physical activity (*p* = 0.031) and marital status (*p* = 0.031) were found; moreover, controls smoked more than IA patients (*p* = 0.007).

### 3.2. PROMIS^®^ Scores between Groups

All participants filled in the previously described questionnaires on psychosocial and gastrointestinal symptoms. Table 2 shows the analysis of each condition’s median t-score (IQR) between groups, through the Mann-Whitney U test, and the number of subjects that reported significant symptoms according to the dichotomization of t-scores. IA patients had increased anxiety, depression, fatigue, sleep disturbance and pain interference, and lower physical function and satisfaction with participation in social roles than controls (each *p* < 0.001). Moreover, gastrointestinal symptoms were more frequent in the patients’ group than in the controls (each *p* < 0.001).

### 3.3. Differences in PROMIS^®^-GI t-Scores among Dichotomized Psychosocial Domains

To investigate the role of psychological and social dimensions, these were dichotomized according to their t-scores, as previously described. A statistical analysis, through the Mann–Whitney U test, was performed to identify differences in the gastrointestinal symptoms t-scores. As shown in Table 3 and Table 4, the psychological dimensions had a significant effect on gastrointestinal symptoms experienced by patients. Belly pain t-score was significantly different in patients experiencing anxiety, sleep disturbances, fatigue, and pain interference than those who did not (each *p* < 0.001), whereas patients with low physical function had higher belly pain (*p* < 0.001). Analysis for constipation showed similar results with *p* < 0.001 for anxiety, sleep disorders, pain interference, and fatigue. A strong difference was also found in disrupted swallowing and all psychosocial symptoms (each *p* < 0.001; pain interference *p* < 0.01).

Both in gas and bloating and nausea and vomiting a significant difference was reported according to the presence or absence of psychosocial domains (each *p* < 0.001, except for satisfaction in social roles *p* < 0.01 for both GI symptoms; and physical function *p* < 0.01 for gas and bloating). Finally, gastroesophageal reflux symptoms were also different in those reporting anxiety, depression, fatigue sleep disturbances, and pain interference (*p* < 0.001).

Then, chi-square tests were carried out to assess the presence of any significant difference between the dichotomized symptoms and the categorical variables (smoking, treatments, and type of IA). No differences in smoking or type of IA were detected; concerning treatment, some differences were found with steroid use among psychological symptoms (Supplementary Tables S2 and S3).

### 3.4. Psychological and Social Impairment Is Associated with Gastrointestinal Symptoms in the Multivariable Logistic Regression Model

As reported in Supplementary Table S4, the multivariable logistic regression model, adjusted for age, sex, BMI, smoking habit, NSAIDs/steroids treatment, and type of IA, showed significant associations between GI symptoms and psychosocial impairment. In more detail, patients experiencing anxiety had higher odds of belly pain (*p* = 0.004), diarrhea (*p* = 0.019), swallowing alterations (*p* = 0.004), bloating/flatulence (*p* < 0.001), and gastroesophageal reflux (*p* = 0.008). Depressive symptoms were associated with belly pain (*p* = 0.035), diarrhea (*p* = 0.017), bloating (*p* = 0.018), and reflux (*p* = 0.01). Patients with fatigue were more likely to report all GI symptoms (belly pain *p* = 0.005; constipation *p* = 0.001; diarrhea *p* = 0.004; disrupted swallowing *p* = 0.001; gas/bloating *p* = 0.006; nausea and vomiting *p* = 0.024 and gastroesophageal reflux *p* = 0.022). Sleep disorders were strongly associated with all the gastrointestinal symptoms, apart from gas and bloating (belly pain *p* < 0.001; constipation *p* = 0.001; diarrhea *p* = 0.001; disrupted swallowing *p* = 0.018; nausea and vomiting *p* = 0.021 and gastroesophageal reflux *p* = 0.005). Pain interference in daily activities was also related to belly pain (*p* = 0.02), constipation (*p* = 0.013), flatulence (*p* = 0.037), nausea/vomiting (*p* = 0.02), and reflux (*p* = 0.035). Conversely, patients with high scores of physical function and satisfaction in social roles were less likely to report constipation (*p* = 0.008 and *p* = 0.025, respectively), diarrhea (*p* = 0.029 and *p* = 0.013, respectively), disrupted swallowing (*p* = 0.016 and *p* < 0.001, respectively) and nausea and vomiting (*p* = 0.004 and *p* = 0.042, respectively). Finally, reporting better physical function also decreased the odds of having belly pain (*p* = 0.007).

## 4. Discussion

In this study, we aimed to assess the prevalence of psychosocial and gastrointestinal symptoms in patients with IA and the associations between these outcomes. Patients with IA report increased impairment in several quality-of-life domains [32,33,34,35]. In our study, IA patients experienced significantly higher psychological and gastrointestinal symptoms than the control population, with more than half of patients showing anxiety, sleep disturbances, fatigue, pain interference, and bloating. Mood disorders, fatigue, physical function alterations, and social life dissatisfaction have been reported widely in the rheumatology setting [36,37,38]. Concerning GI symptoms, fewer data are available. However, the study of Kalkan et al. [39], who surveyed patients with different rheumatologic diseases, demonstrated that more than half of the participants experience abdominal bloating and pain, regurgitation, and heartburn; these results are in line with our findings.

The results of the logistic regression model, adjusted for age, sex, BMI, smoking, NSAIDs, steroids, and type of IA, showed that gastrointestinal symptoms were strongly associated with mood disorders and psychosocial alterations. Specifically, the presence of almost all GI symptoms was affected by anxiety, depression, fatigue, and sleep disturbances (Supplementary Table S4). Therefore, having mood disorders increases the odds of reporting GI complaints. Conversely, physical function and satisfaction in social roles were inversely associated, demonstrating that increased patients’ health perception is related to lower GI symptoms experienced. From this data, it is difficult to determine if the GI symptoms observed in IA patients are a cause or a result of the physiological burden. Conversely, we have here confirmed the GI-brain axis as an important determinant in the quality of life of patients with IA from a clinical perspective.

The relationship between the gut and the brain is a matter of interest in several settings. It is now well known that patients with Irritable Bowel Syndrome have a notable prevalence of mood disorders, especially anxiety, and depression, and that a bidirectional pathway between the two systems is more than likely [40]. Mancina et al. [41] demonstrated that gastrointestinal symptoms are related to psychosocial alterations in clinical remission patients with IBD. It is not surprising that a similar relationship has been found in rheumatic diseases, although the underlying molecular bases have not been clarified.

Gut microbiota alteration has been described in RA [42] and SpA [43], suggesting a role in the pathogenesis and disease course. However, despite the recent advances in the field, no studies have investigated the relationship between gut inflammation or dysbiosis and the brain in the rheumatologic setting. An increasing body of evidence, especially in preclinical models, suggests a bidirectional interplay between the gut-associated immune cells, enteric neuroendocrine cells, gut microbiome, the autonomous nervous system, and the brain. Microbe-derived metabolites and gut-derived molecules interact with the brain modulating its functions [44,45,46].

This new concept has emerged as potentially involved in the pathophysiology of several neurodegenerative diseases and brain disorders, including Autism Spectrum Disorders, Parkinson’s Diseases, and mood disorders [47]. Both probiotics and cognitive behavioral therapy have been reported to modify microbiome compositions and improve depressive symptoms [48,49].

Despite referring to other diseases, these findings should encourage rheumatologists and researchers to address the issue of the gut–brain interplay, even in patients with IA and other rheumatic diseases, to ultimately ensure a comprehensive understanding of the clinical manifestations pathophysiology and assessment of patients’ quality of life. Indeed, despite the vast therapeutic armamentarium available, the management of rheumatic diseases has become more challenging, especially in patients with comorbidities that represent per se a worsening factor. Patients with multi-morbidities including mood disorders (anxiety, depression), sleep disturbances, and fatigue have great impairment of their quality of life which, together with the core disease, leads to poor general and therapeutic outcomes. Likewise, gastrointestinal symptoms, even in the absence of IBD, need to be investigated in patients with IA since they are strongly related and impact daily activities. The concept of integrated medicine is emerging with the aim of filling those unmet needs that are still reported by patients even after a good response to the treatment. A multidisciplinary approach is, therefore, necessary to start treating the patient individually aiming at reducing the impact of IA on the quality of life, and physical and mental health.

However, this study has strengths and limitations. Using single-domain specific questionnaires allows for better detection of symptoms that could be otherwise misinterpreted. Indeed, PROMIS^®^ item banks have demonstrated extensive clinical validity in several chronic conditions [50,51]. Furthermore, an adjusted multivariable model was built to reduce potential bias in the statistical significance. However, since the Italian version of PROMIS^®^-GI scales was not available, the questions were translated by the same authors if the subject was not able to understand. Despite the patients having been recruited consecutively during their scheduled visits, the self-selection of the more high-educated subjects cannot be excluded. This aspect could have variably affected the scores, especially for those patients who required a translation. Moreover, the validation in the US population may not perfectly match the characteristics of the Italian one, for instance in terms of the threshold to consider the patient symptomatic. Indeed, social factors may contribute to influencing both the willingness to complete sincerely the survey and the different perceptions of the symptoms’ burden, leading to a potentially biased score. Thus, further validation studies in the Italian population for the PROMIS^®^-GI item bank are necessary.

Regarding the conditions explored, mood disorders and psychosocial health-related domains are affected by multiple aspects, including personal experiences, socioeconomic and employment status, and the individual capacity to cope with the difficulties. Likewise, reported GI symptoms are based on patients’ perceptions which could not correspond to an actual alteration. It has to be pointed out that these symptoms could be related to other diseases beyond arthritis; in fact, since participants have been seen by a gastroenterologist to systematically exclude any other GI disease (such as GERD, chronic gastritis, treatment-associated gastritis, etc.), but they were assessed only by the anamnesis and clinical records, it cannot be excluded that the reported symptoms derive from other diseases and are not related directly to arthritis. It is also worth noting that although controls were asked if they had comorbidities, including GI diseases, we considered what subjects reported; thus, this could partially explain their high level of GI symptoms. Indeed, as for patients, the control group did not undergo an instrumental evaluation; underlying conditions, that may be linked to the symptoms investigated and that have not been directly reported, cannot be excluded. Moreover, the differences found with patients in terms of demographic and anthropometric factors, need to be further investigated to validate the different reporting of symptoms between groups. A perfectly healthy and matched control group would be necessary to address this issue. In addition, it is worth pointing out that RA and SpA patients present different risks of gastrointestinal symptoms, therefore, it would be essential to design further studies to evaluate the prevalence of these conditions and their association with mood disorders and quality of life, in a separate cohort of RA and SpA.

Moreover, the results were obtained from a sample of two different centers in the same country; thus, a wide generalization, especially in other countries, is uncertain due to the influence of social and cultural factors. A more in-depth investigation of other variables, such as demographic and clinical characteristics, should be carried out to identify other factors associated with the outcomes. In addition, as the nature of a cross-sectional protocol, such as this study, does not provide an estimate of the changes in symptoms’ scores over time, it is necessary to establish whether a specific treatment, including biological agents, steroids, DMARDs, along with psychological or psychiatric therapy, could enhance IA patients’ quality of life and modify all the experienced symptoms accordingly. Finally, in this study, the data only referred to what was reported by the patients. Thus, molecular pathways underpinning this association deserve to be investigated more extensively.

## 5. Conclusions

IA patients experience more significant psychosocial and gastrointestinal symptoms compared with healthy subjects. Anxiety, fatigue, and sleep disturbances are the main determinants of GI impairment. As the symptoms of both dimensions are strictly associated with and impact daily activities, they need to be investigated and become part of general clinical assessment in a multidisciplinary context. Likewise, integrated therapies are mandatory to satisfy patients’ unmet needs. From a clinical point of view, gastrointestinal manifestation should be included in the domain assessed in the rheumatology setting, even without comorbidity of IBD, as it ultimately impacts patients’ quality of life.

## Figures and Tables

**Table 1:** Study Population’s Characteristics.

	Controls (*n* = 238)	IA (*n* = 151)	*p*
**Demographic and Anthropometric**			
Age (years)	53 (38–65)	53 (45–62)	0.931
Sex, n male (%)	147 (61.8)	57 (37.7)	4 × 10^−6^
BMI (Kg/m^2^)	25 (23–28)	27 (24–30)	1.18 × 10^−4^
Smoking, *n* yes (%)	110 (46.2)	49 (32.5)	0.007
Physical Activity, *n* yes (%)	83 (34.9)	37 (24.5)	0.031
High school diploma, *n* yes (%)	137 (57.6)	83 (55.0)	0.615
Marital status, *n* not married (%)	76 (31.9)	33 (21.9)	0.031
Employed, *n* yes (%)	97 (40.8)	65 (43.0)	0.655
**Disease characteristics**			
Rheumatoid Arthritis, *n* (%)	-	47 (31.1)	-
Spondyloarthritis, *n* (%)	-	104 (68.9)	-
Disease duration (years)	-	11 (6–15)	-
Active Disease, *n* yes (%)	-	59 (39.1)	-
ESR (mm/h)	-	12 (6–22)	-
CRP (mg/dl)	-	0.4 (0.2–1.5)	-
**Medications *n* (%)**			
NSAIDs	-	26 (17.2)	-
Steroids	-	20 (13.2)	-
Biological DMARDs	-	101 (66.9)	-
Methotrexate	-	57 (37.7)	-

BMI: Body Mass Index; IA: Inflammatory arthritis; ESR: Erythrocyte sedimentation rate; CRP: C-Reactive Protein; NSAIDs: Non-steroidal anti-inflammatory drugs; biological DMARDs: biological disease-modifying anti-rheumatic drugs.

**Table 2:** PROMIS^®^ scores between groups.

	Controls (*n* = 238)		Inflammatory Arthritis (*n =* 151)		
	Yes	Median t-Score (IQR)	Yes	Median t-Score (IQR)	*p*
Anxiety, *n* (%)	111 (46.6)	49 (43–56)	97 (64.2)	54 (46–60)	<0.001
Depression, *n* (%)	77 (32.4)	46 (38–51)	74 (49.0)	49 (38–57)	<0.001
Fatigue, *n* (%)	85 (35.7)	48 (43–52)	106 (70.2)	55 (49–63)	<0.001
Sleep Disturbance, *n* (%)	62 (26.1)	43 (38–50)	86 (57.0)	51 (46–56)	<0.001
Pain interference, *n* (%)	85 (35.7)	41 (41–52)	126 (83.4)	59 (52–65)	<0.001
Physical Function, *n* (%)	176 (73.9)	60 (49–60)	29 (19.2)	41 (36–48)	<0.001
Satisfaction with participation in social roles, *n* (%)	183 (76.9)	53 (52–60)	54 (35.8)	46 (39–52)	<0.001
Belly pain, *n* (%)	50 (21.0)	34 (34–49)	52 (34.4)	42 (34–54)	0.001
Constipation, *n* (%)	53 (22.3)	37 (37–46)	54 (35.8)	48 (37–53)	<0.001
Diarrhea, *n* (%)	24 (10.1)	40 (40–40)	39 (25.8)	40 (40–50)	<0.001
Disrupted Swallowing, *n* (%)	44 (18.5)	40 (40–46)	48 (31.8)	46 (40–54)	<0.001
Gas and Bloating, *n* (%)	83 (34.9)	45 (38–55)	88 (58.3)	53 (44–58)	<0.001
Nausea and Vomiting, *n* (%)	35 (14.7)	41 (41–46)	55 (36.4)	46 (41–53)	<0.001
Gastroesophageal Reflux, *n* (%)	46 (19.3)	40 (34–49)	47 (31)	45 (37–53)	<0.001

The *p*-values were calculated with the Mann-Whitney U test for non-parametric samples considering the Median t-scores.

**Table 3:** Gastrointestinal symptoms t-scores across dichotomized psychosocial symptoms.

	Belly Pain	*p*	Constipation	*p*	Diarrhea	*p*	Disrupted Swallowing	*p*
Anxiety, no	33.9 (33.9–42.2)	<0.001	38.9 (36.6–49.9)	<0.001	39.9 (39.9–44.1)	0.06	40.3 (40.3–46.0)	<0.001
Anxiety, yes	49.9 (33.9–58.4)		48.5 (41.8–55.3)		39.9 (39.9–51.9)		49.0 (40.3–58.6)	
Depression, no	33.9 (33.9–49.9)	<0.01	45.6 (36.6–51.3)	0.07	39.9 (39.9–45.0)	<0.05	40.3 (40.3–49.0)	0.001
Depression, yes	49.9 (33.9–60.2)		48.1 (36.6–55.7)		39.9 (39.9–53.6)		49.0 (40.3–58.6)	
Fatigue, no	33.9 (33.9–42.2)	<0.001	36.6 (36.6–48.1)	<0.001	39.9 (39.9–44.1)	<0.01	40.3 (40.3–40.3)	<0.001
Fatigue, yes	49.1 (33.9–58.4)		49.7 (41.6–57.1)		39.9 (39.9–51.9)		49 (40.3–58.3)	
Sleep Disturbance, no	33.9 (33.9–47.0)	<0.001	43.2 (36.6–49.7)	0.001	39.9 (39.9–44.1)	<0.01	40.3 (40.3–46.0)	<0.001
Sleep Disturbance, yes	51.1 (33.9–60.2)		49.5 (39.9–57.1)		39.9 (39.9–53.3)		49.0 (40.3–58.3)	
Pain interference, no	33.9 (33.9–36.6)	<0.001	36.6 (36.6–45.3)	<0.001	39.9 (39.9–44.1)	<0.05	40.3 (40.3–43.2)	<0.01
Pain interference, yes	47.0 (33.9–58.4)		48.5 (36.6–55.2)		39.9 (39.9–50.0)		46.0 (40.3–55.3)	
Physical Function, no	47.0 (33.9–58.4)	<0.001	48.5 (36.6–55.2)	<0.01	39.9 (39.9–50.6)	0.001	46.0 (40.3–55.7)	<0.001
Physical Function, yes	33.9 (33.9–42.8)		43.2 (36.6–47.1)		39.9 (39.9–39.9)		40.3 (40.3–40.3)	
Satisfaction with participation in social roles, no	43.3 (33.9–58.4)	<0.05	48.5 (36.6–55.2)	0.053	39.9 (39.9–52.6)	<0.05	49.0 (40.3–59.2)	<0.001
Satisfaction with participation in social roles, yes	33.9 (33.9–50.5)		44.9 (36.6–49.9)		39.9 (39.9–44.1)		40.3 (40.3–46.0)	

The *p*-values were calculated with the Mann-Whitney U test for non-parametric samples.

**Table 4:** Gastrointestinal symptoms t-scores across dichotomized psychosocial symptoms.

	Gas and Bloating	*p*	Nausea and Vomiting	*p*	Gastroesophageal Reflux	*p*
Anxiety, no	45.9 (37.6–54.0)	<0.001	40.6 (40.6–49.6)	0.001	40.7 (33.8–46.2)	0.001
Anxiety, yes	54.6 (47.2–59.2)		49.3 (40.6–55.9)		47.4 (38.8–55.3)	
Depression, no	48.6 (38.7–55.2)	0.001	40.6 (40.6–52.9)	0.001	41.5 (35.2–47.2)	0.001
Depression, yes	54.7 (47.5- 59.7)		49.3 (40.6–55.9)		48.0 (39.1–55.3)	
Fatigue, no	46.6 (37.9–53.3)	<0.001	40.6 (40.6–45.6)	<0.001	39.1 (33.8–43.9)	<0.001
Fatigue, yes	54.7 (43.8–59.2)		49.3 (40.6–55.9)		47.2 (39.1–55.3)	
Sleep Disturbance, no	49.6 (38.7–54.6)	<0.001	40.6 (40.6–49.3)	0.001	39.8 (36.8–46.9)	0.001
Sleep Disturbance, yes	55.2 (44.9–59.8)		49.3 (40.6–55.9)		47.4 (39.1–55.3)	
Pain interference, no	39.10 (37.6–53.0)	<0.001	40.6 (40.6–45.6)	0.001	37.1 (33.8–44.9)	0.001
Pain interference, yes	54.1 (45.3–58.7)		47.5 (40.6–55.9)		46.3 (38.2–54.1)	
Physical Function, no	53.8 (43.8–58.7)	<0.01	47.5 (40.6–55.9)	0.001	46.3 (37.1–54.1)	<0.01
Physical Function, yes	45.3(37.6–54.9)		40.6 (40.6–45.6)		41.5 (33.8–46.0)	
Satisfaction with participation in social roles, no	54.6 (43.8–59.2)	<0.01	49.3 (40.6–55.9)	<0.01	46.0 (37.1–53.6)	0.455
Satisfaction with participation in social roles, yes	48.2 (39.1–54.8)		40.6 (40.6–49.6)		43.9 (36.9–51.8)	

The *p*-values were calculated with the Mann-Whitney U test for non-parametric samples.

## Data Availability

The data presented in this study are available on request from the corresponding author. The data are not publicly available due to personal information in the database.

## Supplementary Materials

The following supporting information can be downloaded at: https://www.mdpi.com/article/10.3390/jcm12093248/s1, Supplementary Table S1: Disease activity scores; Supplementary Table S2: Categorical variables dichotomized across psychosocial symptoms; Supplementary Table S3: Categorical variables dichotomized across gastrointestinal symptoms; Supplementary Table S4: Multivariable logistic regression analysis.
