# Supplementary material for: Gastrointestinal Symptoms Impact Psychosocial Function and Quality of Life in Patients with Rheumatoid Arthritis and Spondyloarthritis: A Cross-Sectional Study
Source: J Clin Med. 2023 May 1;12(9):3248. doi: 10.3390/jcm12093248 (PMC10179391; doi:10.3390/jcm12093248)
Supplement: Supplementary file 1 [file jcm-12-03248-s001.zip › Supplementary Table S1 Disease Activity Scores.pdf]

Supplementary Table S1. Disease activity scores

**DAS28-CRP**	
**Component**	**Measure**
Tender joint count	0-28
Swollen joint count	0-28
Patient's Global Health - Visual analog scale	0-100
C-reactive protein (CRP)	mg/L
**DAPSA28-CRP**	
**Component**	**Measure**
Tender joint count	0-28
Swollen joint count	0-28
Pain – Visual analog scale	0-100
Patient's Global Health - Visual analog scale	0-100
C-reactive protein (CRP)	mg/L
**ASDAS**	
Back pain	0-10
Peripheral pain/swelling	0-10
Duration of morning stiffness	0-10
Patient global assessment of disease activity	0-10
C-reactive protein (CRP)	mg/L

DAS28-CRP: Disease Activity Score 28 with C-Reactive Protein
DAPSA28: Disease Activity Index for PSoriatic Arthritis 28 with C-Reactive Protein
ASDAS: Ankylosing Spondylitis Disease Activity Score
