# Supplementary material for: Gastrointestinal Symptoms Impact Psychosocial Function and Quality of Life in Patients with Rheumatoid Arthritis and Spondyloarthritis: A Cross-Sectional Study
Source: J Clin Med. 2023 May 1;12(9):3248. doi: 10.3390/jcm12093248 (PMC10179391; doi:10.3390/jcm12093248)
Supplement: Supplementary file 1 [file jcm-12-03248-s001.zip › Supplementary Table S2 Categorical variables dichotomized across psychosocial symptoms.pdf]

Supplementary Table S2 Categorical variables dichotomized across psychosocial symptoms

	Anxiety		*p*	Depression		*p*	Fatigue		*p*	Sleep disturbances		*p*	Pain interference		*p*	Physical Function		*p*	Satisfaction with participation in social roles		*p*
	Y	N		Y	N		Y	N		Y	N		Y	N		Y	N		Y	N	
SMOKING																					
Y	35	14	0.201	20	29	0.083	37	12	0.323	29	20	0.701	43	6	0.323	11	38	0.483	18	31	0.863
N	62	40		45	57		69	33		57	45		83	19		18	84		36	66	
IA																					
RA	34	13	0.163	28	19	0.081	38	9	0.054	30	17	0.251	43	4	0.074	6	41	0.177	17	30	0.944
SpA	63	41		46	58		68	36		56	48		83	21		23	81		37	67	
NSAIDs																					
Y	16	10	0.752	12	14	0.749	19	7	0.724	13	13	0.431	21	5	0.687	5	21	0.997	9	17	0.893
N	81	44		62	63		87	38		73	52		105	20		24	101		45	80	
STEROIDS																					
Y	15	5	0.281	15	5	0.013	17	3	0.120	14	6	0.206	20	0	0.032	1	19	0.083	3	17	0.038
N	82	49		59	72		89	42		72	59		106	25		28	103		51	80	
METHOTREXATE																					
Y	41	16	0.161	32	25	0.172	74	27	0.241	32	25	0.875	55	2	0.001	7	50	0.093	17	40	0.236
N	56	38		42	52		32	18		54	40		71	23		22	72		37	57	
BIOLOGIC AGENTS																					
Y	59	42	0.034	48	53	0.605	45	12	0.067	57	44	0.855	83	18	0.552	17	84	0.293	34	67	0.445
N	38	12		26	24		61	33		29	21		43	7		12	38		20	30	

Categorical variables dichotomized across psychosocial symptoms were compared using the chi-square test. *p*-value < 0.05 was considered to be significant. Abbreviations: IA: Inflammatory Arthritis; RA: Rheumatoid Arthritis; SpA: Spondyloarthritis; NSAIDs: non-steroidal anti-inflammatory drugs
