# Supplementary material for: Gastrointestinal Symptoms Impact Psychosocial Function and Quality of Life in Patients with Rheumatoid Arthritis and Spondyloarthritis: A Cross-Sectional Study
Source: J Clin Med. 2023 May 1;12(9):3248. doi: 10.3390/jcm12093248 (PMC10179391; doi:10.3390/jcm12093248)
Supplement: Supplementary file 1 [file jcm-12-03248-s001.zip › Supplementary Table S3 Categorical variables dichotomized across gastrointestinal symptoms.pdf]

Supplementary Table S3 Categorical variables dichotomized across gastrointestinal symptoms

	Belly Pain		*p*	Constipation		*p*	Diarrhea		*p*	Disrupted swallowing		*p*	Gas and Bloating		*p*	Nausea and Vomiting		*p*	Gastroesophageal Reflux		*p*
	Y	N		Y	N		Y	N		Y	N		Y	N		Y	N		Y	N	
SMOKING																					
Y	18	31	0.680	21	28	0.207	15	34	0.352	19	30	0.201	28	21	0.845	22	27	0.134	19	30	0.159
N	34	68		33	69		24	78		29	73		60	42		33	69		28	74	
IA																					
RA	18	29	0.502	18	29	0.662	14	33	0.455	20	27	0.056	32	15	0.100	19	28	0.492	14	33	0.811
SpA	34	70		36	68		25	79		28	76		56	48		36	68		33	71	
NSAIDs																					
Y	7	19	0.375	9	17	0.893	8	18	0.527	11	15	0.205	15	11	0.947	9	17	0.833	10	16	0.375
N	45	80		45	80		31	94		37	88		73	52		46	79		37	88	
STEROIDS																					
Y	6	14	0.654	7	13	0.939	7	13	0.314	5	15	0.484	11	9	0.750	8	12	0.721	6	14	0.907
N	46	85		47	84		32	99		43	88		77	54		47	84		41	90	
METHOTREXATE																					
Y	20	37	0.896	19	38	0.628	17	40	0.382	20	37	0.498	35	22	0.544	23	34	0.435	22	35	0.123
N	32	62		35	59		22	72		28	66		53	41		32	62		25	69	
BIOLOGIC AGENTS																					
Y	36	65	0.657	38	63	0.497	25	76	0.668	32	69	0.969	62	39	0.271	41	60	0.130	30	71	0.591
N	16	34		16	34		14	36		16	34		26	24		14	36		17	33	

Categorical variables dichotomized across gastrointestinal symptoms were compared using the chi-square test. *p*-value < 0.05 was considered to be significant. Abbreviations: IA: Inflammatory Arthritis; RA: Rheumatoid Arthritis; SpA: Spondyloarthritis; NSAIDs: non-steroidal anti-inflammatory drugs
